# Fecal Bile Salts and the Development of Necrotizing Enterocolitis in Preterm Infants

**DOI:** 10.1371/journal.pone.0168633

**Published:** 2017-01-03

**Authors:** Christian V. Hulzebos, Anne G. J. F. van Zoonen, Jan B. F. Hulscher, Trijntje E. Schat, Elisabeth M. W. Kooi, Martijn Koehorst, Renze Boverhof, Paul F. M. Krabbe, Albert K. Groen, Henkjan J. Verkade

**Affiliations:** 1 Department of Pediatrics, Division of Neonatology, Beatrix Children’s Hospital, University of Groningen, University Medical Center Groningen, Groningen, the Netherlands; 2 Department of Pediatric Surgery, University of Groningen, University Medical Center Groningen, Groningen, the Netherlands; 3 Department of Pediatrics, Division of Hepatology/Gastroenterology, Beatrix Children’s Hospital, University of Groningen, University Medical Center Groningen, Groningen, the Netherlands; 4 Department of Epidemiology, University of Groningen, University Medical Center Groningen, Groningen, the Netherlands; 5 Amsterdam Diabetes Center, Department of Vascular Medicine, Academic Medical Center, Amsterdam, the Netherlands; Children's Hospital of Los Angeles, UNITED STATES

## Abstract

**Background:**

Intestinal bile salts (BSs) may be implicated in NEC development. We hypothesized that fecal BS levels are higher in preterm infants at risk for NEC.

**Methods:**

We compared the composition and concentration of fecal BSs in ten preterm infants who developed NEC (Bell’s Stage ≥ II) with twenty matched control infants without NEC. Conjugated and unconjugated fecal BSs were measured after birth (T1) and twice prior to NEC (T2, T3). Data are presented as medians and interquartile ranges.

**Results:**

GA and BW were similar in all preterms: ~27^+4^ weeks and ~1010 g. Age of NEC onset was day 10 (8–24). T1 was collected 2 (1–3) days after birth. T2 and T3 were collected 5 (5–6) days and 1 (0–2) day before NEC or at corresponding postnatal ages in controls. The composition of conjugated BSs did not differ between the two groups. Total unconjugated BSs were 3-fold higher before NEC compared to controls at corresponding ages (0.41 μmol/g feces (0.21–0.74) versus 0.14 μmol/g feces (0.06–0.46), *p* < 0.05).

**Conclusion:**

Fecal BS concentrations are higher in preterm infants who develop NEC compared to infants without NEC. Further study is needed to determine the predictive value of fecal BSs in the development of NEC.

## Introduction

It remains unpredictable whether or not a preterm infant will develop NEC, despite well-characterized epidemiological and clinical risk factors such as low gestational age (GA), low birth weight (LBW), formula feeding, and antibiotic treatment [1]. Early symptoms of NEC are often non-specific, as are laboratory parameters and radiological examinations. Therefore, finding parameters that could identify preterm infants at risk of NEC is of great clinical importance.

It is thought that the accumulation of intestinal bile salts (BSs) is implicated in the development of NEC. BSs are the main organic constituents of bile and act as biological detergents that facilitate intestinal absorption of dietary fats. The so-called primary BSs, i.e. cholate and chenodeoxycholate, are synthesized from cholesterol in the human liver and are conjugated with either the amino acid glycine or taurine. The synthesis of BSs already starts *in utero*, and BSs can be detected in meconium [2]. Intestinal BS re-uptake occurs mainly in the distal ileum, i.e. the intestinal segment predominantly affected in NEC. The incidence of NEC is highest in formula-fed preterm infants. In comparison to term infants it has been shown that BS levels and BS composition in preterm infants are different and levels of secondary and more hydrophobic BSs are higher in feces of formula-fed infants than in breast-fed infants [3,4]. Intraluminal accumulation of hydrophobic BSs can result in intestinal epithelial damage similar to the histopathological findings in NEC [5]. Previously, biliary hypersecretion of BSs has been shown to promote NEC development in mice, whereas intraluminal capture of BS in the intestine by binding agents (e.g. cholestyramine) has been shown to mitigate NEC in a rodent model [6]. Intestinal damage occurs when high concentrations of intraluminal intestinal BS lead to intracellular intestinal accumulation. The essential role of intestinal BS transport in the development of NEC has been convincingly demonstrated: NEC is attenuated in mice deficient in apical sodium-dependent bile salt transporter (ASBT), a protein involved in intestinal BS uptake, and in rats treated with an ASBT inhibitor [6]. In agreement with the postulated role of increased BS uptake in the distal small intestine, ASBT expression (mRNA and immunohistochemistry) was increased in intestinal samples of preterm infants with NEC in comparison to ileal samples taken during surgery on preterm infants with other diseases [6]. Furthermore, BSs have been shown to decrease ileal mucin production more profoundly in immature than in older ileums, and alter the intestinal mucus layer which might compromise the intestinal barrier function [7].

The foregoing data support the putative role of high intestinal BSs in NEC development. We hypothesized that high levels of fecal BSs might serve as early parameters to identify infants with imminent NEC development. Prospectively, we determined fecal BS concentrations and compositions in preterm infants at risk for NEC. Subsequently, we compared BS parameters of infants who later developed NEC with GA and BW-matched infants who did not develop the disease.

## Materials and Methods

### Ethics statement

We obtained written, informed parental consent in all cases. The study was approved by the ethics review board of University Medical Center Groningen.

### Patients

This study was a case-control study and part of a large, prospective observational cohort registered with the Dutch Trial Registry (trialregister.nl; identifier: NTR4153). The study was performed in the NICU of the Beatrix Children’s Hospital of the University Medical Center Groningen (UMCG) between October 2012 and February 2014. For the purpose of the cohort study, preterm infants with a GA below 30 weeks, a BW below 1000 grams, or a combination of a GA below 32 weeks and a BW below 1200 grams were eligible for enrollment within the first 48 hours after birth. Exclusion criteria consisted of congenital intestinal diseases or abdominal wall defects. One hundred infants were included in the cohort study. For the present study, we selected all infants with NEC (n = 10) and matched two controls from the same cohort to each infant who developed NEC, using GA and BW, in descending order of importance, allowing for a maximum deviation of 10%.

#### Clinical data

We collected all data prospectively. Neonatal data included gender, GA, BW, twin or singleton, antenatal treatment with steroids, preterm prelabor rupture of membranes >24 hours, Apgar scores at 1, 5, and 10 minutes, antibiotic treatment during first 48 hours after birth, prolonged antibiotic treatment (> 48 hours after birth), hemodynamically significant patent ductus arteriosus (hsPDA), ibuprofen treatment for hsPDA, time to onset of enteral feeding and type of enteral feeding. In addition, we recorded the need for mechanical ventilation, inotropics, fluid resuscitation, and red-blood cell transfusion during the study period as well as mortality data. Data are in S1 File.

#### Feeding strategy

Enteral feeding consisted of own mother’s milk, preterm formula, or a combination if own mother's milk was not (sufficiently) available in 21 infants. Nine patients (1 NEC and 8 controls) had been included in the Early Nutrition Study (trialregister.nl; identifier: NTR3225), a RCT comparing effects of pasteurized donor milk or preterm formula during the first 10 days of life in addition to mother's own milk in very low birth weight infants (< 1500 g.) [8]. Enteral feeding was started in all patients according to the local guideline. In short, preterms with a birth weight < 1250 g. were fed 12 times per 24 hours, and preterm infants > 1250 g. were fed 8 times daily. Feeding volume was started at 15–20 mL per kg and was increased daily with the same volume. Breast milk fortifier (Nenatal BMF^®^, Nutricia, Zoetermeer, the Netherlands) was added after the first week when feeding volume was minimally 80 mL/kg/day. Parenteral nutrition was started immediately after birth; amino-acids from the date of birth and fat emulsions thereafter.

#### Primary outcome

The primary outcome of this study was a combination of fecal BS concentrations and composition in preterm infants who did, or did not, develop NEC. NEC diagnosis was defined by the presence of either pneumatosis intestinalis or portal venous gas, or both according to the modified Bell’s staging criteria (Bell’s Stage ≥ II). The diagnosis was established by the attending radiologist and later confirmed independently by five neonatology consultants. We defined the time of NEC onset as the first abdominal X-ray after clinical suspicion of the disease. All preterm infants with NEC were treated conservatively with nil per os, gastric decompression, and broad-spectrum antibiotics until radiographic signs of NEC resolved and clinical signs stabilized. Surgery was performed when a perforation was suspected or when the infant deteriorated clinically despite conservative treatment.

### Collection of fecal samples

Fecal samples were collected from the diaper in sterile tubes within 48 hours after birth and subsequently twice weekly on every first and fourth day calculated from birth, until either NEC Bell’s Stage ≥ II occurred, or the infant was transferred to another hospital, or reached the age of 36 days. If the infant did not produce feces on the predetermined day of feces sampling, the next feces produced was collected (up op two days after the predetermined sampling day). In controls, selection of feces samples was adjusted according to the closest corresponding time point of feces samples from the infants who ultimately developed NEC. Fecal samples were stored at -80°C until batch analysis of unconjugated and conjugated BSs.

#### Fecal bile salt analysis

Fecal BS concentrations were measured in an aliquot of freeze-dried homogenized feces and determined by capillary gas chromatography (GC) as methylester-trimethylsilyl derivatives on a Hewlett Packard gas chromatograph (HP 5880 A) as previously described [9]. GC methodology provides reliable quantitative information on BS species, irrespective of the conjugation status, as the standard operating procedure includes a deconjugation procedure. Unconjugated BS concentrations are expressed in μmol/g dry weight. We measured the levels of cholate (C), chenodeoxycholate (CDC), deoxycholate (DC), hyocholate (HC), and hyodeoxycholate (HDC). Total fecal BS levels were calculated as the sum of all measured individual species. Because more BS species exist, the absolute level of total BSs should be read as total *measured* BSs. The lower limit of detection of unconjugated BSs varies between 0.3 and 0.5 nmol dry weight feces, but is slightly dependent on the BS species (C: 0.4 nmol, CDC: 0.4 nmol, DC: 0.3 nmol, HC: 0.4 nmol, HDC: 0.5 nmol). Fecal conjugated BS composition was measured by liquid chromatography-tandem mass spectrometry (LC-MS/MS). This system consists of a Shimadzu liquid chromatography system (Shimadzu, Kyoto, Japan) coupled to a Sciex API-3200 triple quadrupole mass spectrometer with an electrospray ionization (ESI) source (Sciex, Framingham, Massachusetts, USA). In short, about 50 mg of freeze-dried homogenized feces was weighed exactly and extracted with 2 mL of alkaline methanol for two hours at 80°C. After cooling down to room temperature, 8 mL of demineralized water was added and the tubes were vortexed. After adding 250 μL internal standard solution to 1 mL of sample solution, the samples were prepared for analysis using solid phase extraction (SPE). After SPE, the samples were dried using N_2_ at 40°C, and the samples were redissolved in 500 μL 50% methanol. Finally, the samples were filtered with 0.2 μm spin filters. Ten μL of sample was injected into the LC-MS/MS system. The BS species we evaluated were glycocholate (GC), taurocholate (TC), glycochenodeoxycholate (GCDC), and taurochenodeoxycholate (TCDC). Data are in S2 File and in S3 File. In theory, the LC-MS/MS method may be used to detect both the conjugated and unconjugated BA. However, we identified two important limitations for BS measurements in feces by LC-MS/MS: i) In feces there were abundant interfering compounds in the chromatograms. This feature made it hard, and for some BS even impossible to reliably obtain quantitative information of BS concentrations. We only could determine concentrations of a specific BS when its peak in the chromatogram and the corresponding internal-standard were sufficiently separated and not disturbed by apparently interfering compounds. ii) The linear range in which BSs can be accurately detected was much smaller with the LC-MS/MS methodology compared to the GC methodology. For the LC-MS/MS method samples had to be diluted extensively, which may have reduced levels of some of the BSs even below the detection limit.

### Statistical analysis

All data of fecal BSs are presented as medians and interquartile ranges (IQR) unless specified otherwise. Either odds ratios (and 95% confidence intervals (CIs)) or the Mann-Whitney test was used to analyze differences in clinical characteristics, as appropriate. The Median Test (one-tailed) was used to compare levels of fecal unconjugated BS concentrations and of percentages of conjugated BS profiles between the two groups at the three different points in time (independent variables). The Wilcoxon signed Rank Test (two-tailed) was used to compare median levels of fecal BS concentrations within subgroups (dependent variables). Cut-off values for NEC prediction were investigated with receiver operating characteristics (ROC) analyses. The level of significance for statistical analyses was set at *p* < 0.05. All analyses were performed using SPSS 22.0 software for Windows (IBM SPSS Statistics 22, IBM Corporation, Armonk, New York, USA).

## Results

### Patient characteristics

Ten preterm infants developed NEC during the study period at a median age of 10 (4–30) days after birth. Bell’s Stage II occurred in two infants and Bell’s Stage III in eight. Seven infants underwent surgery. In one infant surgery was indicated but not performed due to the infant’s poor clinical condition. Three out of the ten infants who developed NEC died as a consequence of the disease. Table 1 shows that demographic and clinical characteristics were not significantly different between the infants who did, or those who did not, develop NEC.

**Table 1:** Patient characteristics.

	NEC (n = 10)	No NEC (n = 20)	OR (95%-CI)
GA, weeks	27.5 (24.6–29.4)	27.6 (25.3–29.9)	
BW, grams	1010 (775–1630)	1013 (615–1735)	
Male: Female	5:5	11:9	
Apgar score 10’	8 (4–10)	8 (7–10)	
Postnatal age NEC (days)	9.5 (4–30)	NA	
DCDA gemelli (%)	30	30	1 (0.19–5.24)
Antenatal steroids (%)	90	90	1 (0.08–12.56)
PPROM (%)	70	35	4.3 (0.85–22.23)
Mechanical ventilation (%)	70	55	1.9 (0.38–9.59)
hsPDA—NSAID (%)	40	35	1.24 (0.26–5.91)
Enteral feeding < 24 hrs (%)	100	100	
Type of feeding			
MM	2	4	1 (0.15–6.67)
MM+DM	0	2	0 (0-∞)
MM+PF	5	13	0.54 (0.11–2.51)
PF	3	1	8.14 (0.72–91.89)
Antibiotics first 48 hrs (%)	90	85	1.59 (0.14–17.56)
LOS (%)	70	35	4.3 (0.84–22.23)
Saline infusion (%)	60	45	1.83 (0.39–8.57)
Inotropes (%)	40	10	6 (0.87–41.4)
RBC transfusion (%)	80	65	2.15 (0.36–13.05)
Mortality (%)	30	10	3.86 (0.53–28.24)
Postnatal day of death	27 (6–33)	8.5 (6–11)	

Data are expressed as median (range) or as numbers unless specified otherwise. DCDA, dichorionic diamniotic; PPROM, preterm prelabour ruptures of membranes > 24 h.; hsPDA, hemodynamically significant patent ductus arteriosus; NSAID, nonsteroidal anti-inflammatory drug; MM, mother’s milk; PF, preterm formula, DM, donor milk; LOS, late onset sepsis; RBC, red blood cell; N.A., not applicable.

### Feces sampling

A total of 76 fecal BS samples were available for analysis of preterm infants: 25 samples of infants who ultimately developed NEC and 51 samples of control infants. Unconjugated BSs could be measured in 59 samples (24 NEC and 35 controls), and conjugated BSs could be measured in 69 samples (22 NEC and 47 controls). In all infants, the first feces sample (T1) was collected at a median of 2 days after birth (IQR 1–3). In all infants, the two consecutive samples taken immediately prior to the diagnosis of NEC, T2 and T3, were collected at a median of 5 days (IQR: 5–6) and 1 day (IQR: 0–2) before onset of NEC, respectively. In NEC infants, the last feces sample was collected at a median (IQR) of 1.5 (0–3) day before NEC occurred. In controls, median (IQR) day of life (DOL) at T2 was 5 (4–21.5) days, compared to 7.5 (4–20) in the NEC infants (NS). Median (IQR) DOL at T3 was 11.5 (8–24) in controls and 12 (8–12) in NEC infants (NS).

### Total fecal bile salts

#### Total unconjugated bile salts

**Fig 1:**
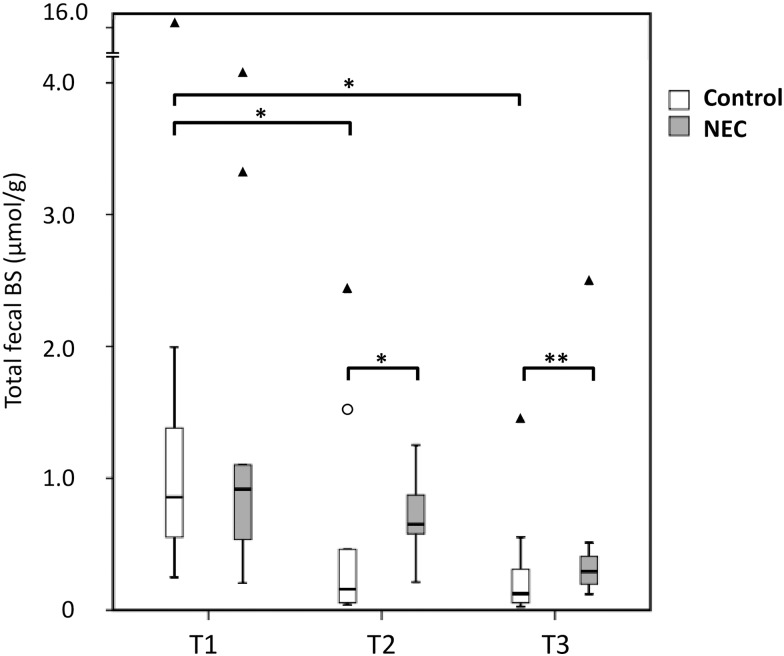
Box plot of total BS concentrations. The median is marked by the horizontal line in the central box. The boxes are limited by the 25th and 75th percentiles. The whiskers (┴) represent the lowest and highest total BS within 1.5 interquartile distance below or above the box. Outliers (○) represent BS concentrations between 1.5 and 3 interquartile distances below or above the box. Extremes (▲) represent BS concentrations more than 3 interquartile distances above the box. Feces was collected at three different points in time; within three days after birth (T1), five to six days prior to NEC (T2), zero to two days prior to NEC (T3). Total BS concentration: sum of all individual BS concentrations, irrespective of the conjugation status, detected with gas chromatography and expressed in μmol/g dry weight feces.*: *p* < 0.05, **: *p* < 0.1 (T3: *p* = 0.07).

Total BS concentrations were not significantly different in the first feces samples after birth (T1) between the NEC group and the controls (Fig 1). At T2, which corresponded to five days prior to NEC, total unconjugated BSs were four times higher in the NEC group than in the controls (0.65 μmol/g feces (0.49–0.97) versus 0.16 μmol/g feces (0.48–0.99), *p* = 0.02). In the feces samples at T3, one day prior to NEC, total unconjugated BSs were two-and-a half times higher in the NEC group than in the controls (0.29 μmol/g feces (0.19–0.46) versus (0.13 μmol/g feces (0.05–0.37), *p* = 0.07). When the fecal samples of T2 and T3 were analyzed together, we found that total unconjugated BSs were significantly increased in infants developing NEC (median 0.41 μmol/g feces (0.21–0.74) versus 0.14 μmol/g feces (0.06–0.46), *p* = 0.03).

We observed no statistically significant decrease in total fecal unconjugated BSs between any of the time points in the infants developing NEC. The decrease in total BSs was statistically significant in the controls between T1 (0.86 μmol/g feces (0.55–1.41) and T2 (0.16 μmol/g feces (0.05–0.99), *p* = 0.01, Fig 1), and also between T1 and T3 (-85%, *p* < 0.05).

#### Unconjugated bile salt species

**Fig 2:**
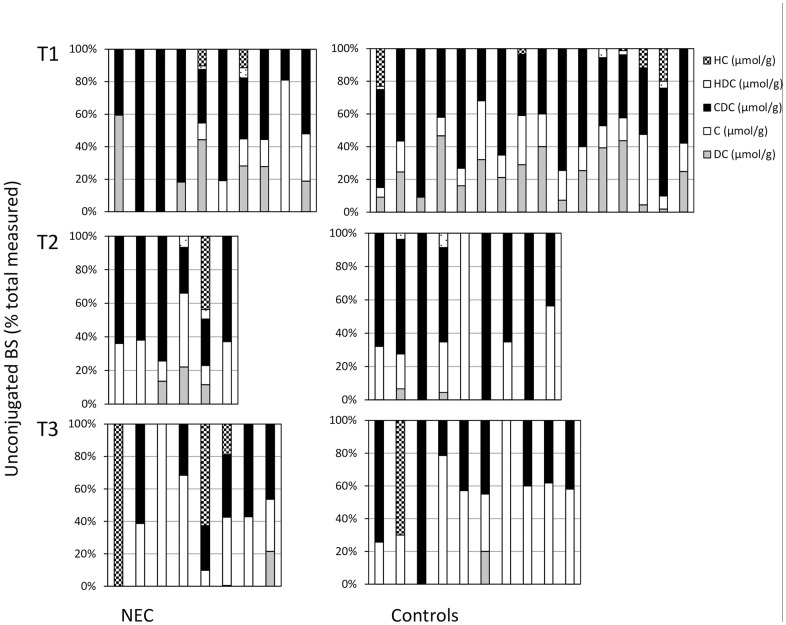
Percentage of specific bile salts (BSs) at three time points. Specific BSs were measured at three different time points before NEC occurred or in a matched control sample with GC methodology as explained in the Methods section. Specific BS (hyocholate (HC), hyodeoxycholate (HDC), chenodeoxycholate (CDC), Cholate (C), and deoxycholate (DC)) are given as percentage of the total measured BSs.

Of unconjugated BSs, chenodeoxycholate (CDC) was the only unconjugated BS species that showed a trend toward significance between the NEC group and the controls. Unconjugated CDC concentrations were higher in the infants developing NEC in the week prior to NEC (0.18 μmol/g feces (0.13–0.43) versus 0.08 μmol/g feces (0.03–0.28), *p* = 0.06). Fig 2 shows the specific BS as a percentage of the total measured BS concentration at three different time points before NEC occurred or in a matched control sample. The primary BSs cholate (C) and chenodeoxycholate (CDC) were abundant at all time points in preterm infants who developed NEC and also in matched control infants. HC and/or HDC, that are rare in humans, were present in 7 of 24 (29%) samples of NEC patients and in 9 of 35 (26%) samples of controls (NS). In the last feces sample before NEC occurred, HC was present in 3 of 8 samples in NEC infants, and in 1 of 10 control samples (NS).

#### NEC prediction with receiver operating characteristic data

**Fig 3:**
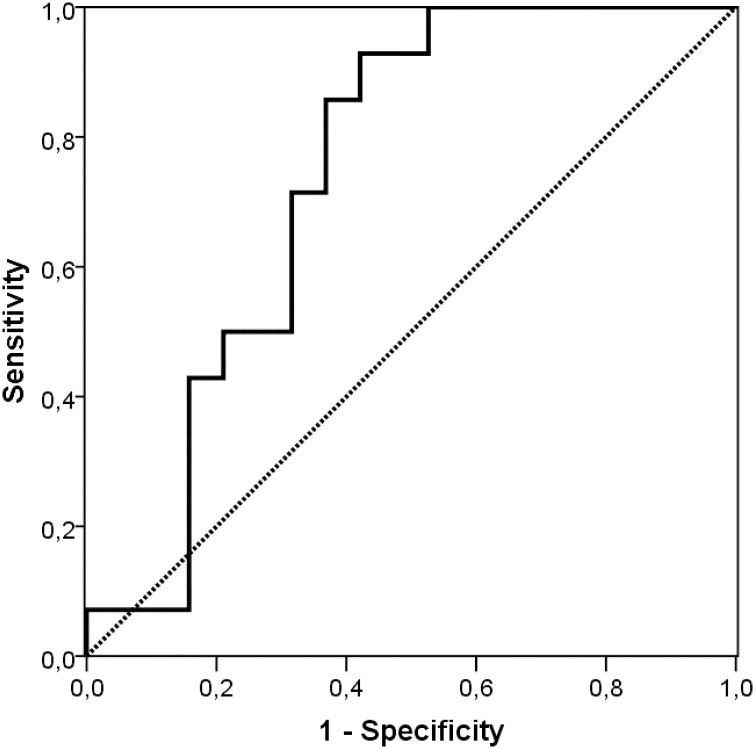
Receiver operating characteristic (ROC) curve of total fecal BS concentrations in the week prior to NEC onset.

The ROC curve of total BSs at T2 and T3 illustrated that total unconjugated BS > 0.13 μmol/g feces tended to predict NEC with 93% sensitivity and 47% specificity (area under curve (AUC) 0.74. 95% CI 0.57–0.91; *p =* 0.02, Fig 3).

#### Fecal conjugated bile salts

**Fig 4:**
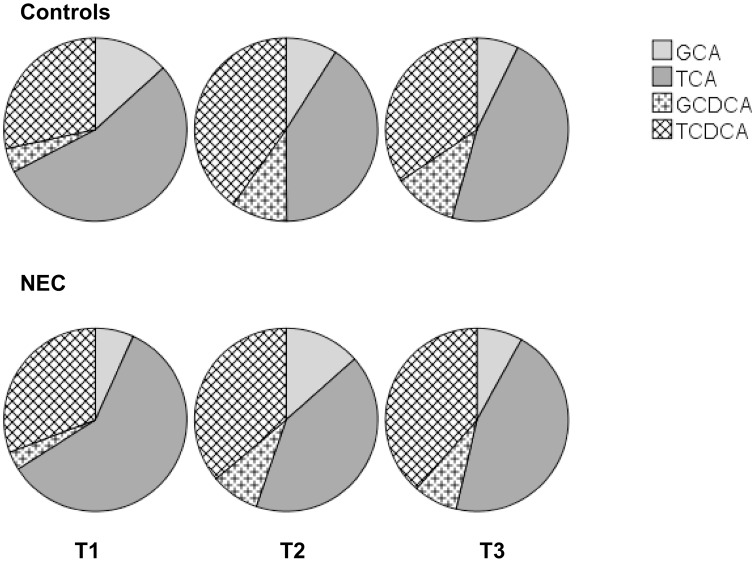
Composition of fecal conjugated bile salts. Feces were collected at three different points in time: within three days after birth (T1), four to six days prior to NEC (T2), zero to two days prior to NEC (T3). Fecal conjugated bile salt species were measured with LC-MS/MS as described in the Methods section.

Fig 4 shows the composition of conjugated BSs in both the NEC group and the controls. We found no statistically significant differences in fecal conjugated BS composition at the three sampling points between preterm infants who developed NEC and controls.

## Discussion

Our aim was to determine whether fecal BS analysis could, potentially, provide an early marker for the development of NEC in preterm infants. We demonstrated a significantly higher level of total fecal BSs in the week before NEC started to develop compared to corresponding samples in GA and BW matched controls. Yet, the differences in the composition of conjugated fecal BSs at any of the three time points were not statistically significant between the NEC group and the controls. The difference in fecal BS could also not be attributed to major differences in specific, individual unconjugated BS. Apparently, the decrease was quantitative more that qualitative, and the variation in individual BS concentrations precluded statistical significance. Taking into consideration the sensitivity and specificity of fecal BSs for the development of NEC, it seems impossible at this stage to predict the onset of NEC using fecal BS analysis in preterm infants.

Our results are in accordance with previous analyses of total BS concentrations in neonates, i.e. with respect to the highest levels for total BSs in meconium [10]. In our study, apart from unconjugated BSs, we also detected conjugated BS species in the first available fecal samples, i.e. within the first three days after birth. In analogy to previous data, including biliary and duodenal BSs in preterm and term infants, taurine-conjugated BSs predominated [11,12]. BS conjugation increases hydrophilicity, which promotes aqueous solubility at intestinal pH, and if intravascular, it facilitates renal excretion, thus minimizing the membrane damaging properties of the more hydrophobic unconjugated bile salts [13]. In the intestines, particularly in the colon, conjugated cholate and chenodeoxycholate may undergo deconjugation, and can be substrates for dehydroxylation by bacterial flora, resulting in deoxycholate and lithocholate, which are more toxic. Animal data, and preliminary human data as well, have implicated intestinal BSs in the development of NEC [5]. Bile salt levels in intestinal tissue are increased in rodents with experimentally induced NEC and reduction of intestinal BS reabsorption by enteral administration of cholestyramine decreases both its rate and severity [5]. Moreover, rate as well as severity of experimental NEC were reduced in apical sodium-dependent bile acid transporter (ASBT) knockout mice, and also in rats in which ASBT was pharmacologically inhibited [6]. Finally, more severe NEC was demonstrated in genetically manipulated mice which hypersecrete biliary BSs, resulting in increased concentrations of intestinal BSs. In accordance, increased mRNA levels of ASBT in ileal tissue of preterm infants with NEC is suggestive of increased BS uptake into enterocytes [6]. In our study, total unconjugated BSs in feces decreased at a slower rate before NEC development in comparison to controls. Similar to our data of higher total BSs levels in feces of preterm infants prior to NEC, intestinal luminal BSs levels in rodents subjected to experimental NEC were higher than in control animals [14]. To some extent, our findings are also in agreement with preliminary data on serial total fecal BS excretion in two preterm neonates with comparable GA and BW, i.e. 29 weeks and 1050 grams, who developed NEC (personal communication with professor M.D. Halpern, University of Arizona). Increases of total fecal BSs and higher coefficients of variation were demonstrated in these preterm infants. On the basis of previous animal and human data, higher fecal BS concentrations could be anticipated upon after birth in preterm infants with imminent NEC. It is tempting to speculate that this transient failure to decrease BSs coincides with increased levels of intraenterocytic BSs of the preterm intestine. A prerequisite would be sufficient activity or even upregulation of ASBT in the immature intestine, in which the negative feedback of intestinal BSs on ASBT, and on intestinal BS uptake, is abnormal or does not yet occur. Whether intestinal microbiota affect the expression of ASBT in preterm infants, as was previously reported in mice, is unknown [15].

We acknowledge several limitations to our study and results. Obviously, due to its size, our sample lacked the power to discriminate between a normal and a slow albeit “NEC-predisposing” decay pattern of fecal BSs. A substantial larger sample size (i.e., of at least 50 NEC infants) would be required for sufficient power. Secondly, measurement of fecal BSs on one particular day does not allow one to predict whether and if so when NEC will occur: serial measurements from birth until NEC onset should be obtained to assess the decay of fecal BSs. Thirdly, higher levels of fecal BSs, or a slower decrease in fecal BSs, may help to identify preterm infants with an increased risk of developing NEC, but it remains unknown how we can reduce the risk of NEC in these particular infants. Finally, no data on the fecal microbiome, that would affect bile salt composition, were provided.

The applied GC methodology allows for reliable and fast quantification of many BSs in a way that may more or less be routinely incorporated in clinical care for preterm infants. Yet, we could not detect either conjugated or unconjugated BS in some feces samples. Upon its consistency upon reanalysis, it is tempting to speculate that—apart from any methodological issues—variations in the developing microbiome of the immature intestine in preterm infants is responsible for these variations. We did not choose any (un)conjugated at forehand. Rather, these particular BS species appeared to be present upon analysis by our methodology. Interestingly, HC and HDC are usually not detected in appreciable amounts in (adult) humans. The presence of these BS species in some of the samples is not clarified. The observation that they are found both in NEC and in non-NEC feces pleads against an important pathophysiological role in the development of NEC.

Despite predictive clinical risk factors NEC remains a multifactorial disease with rapid unpredictable onset of intestinal damage for which powerful predictive biochemical markers are urgently needed, but these are, as yet, unavailable [16,17]. The slower decay of fecal BSs provides support for altered BS metabolism in the development of NEC in humans. Further study, with serial measurements of fecal BSs in a large number of high-risk infants, is needed to firmly establish the role of fecal BSs in the prediction of NEC in preterm infants.

## Conclusion

In preterm infants who develop NEC, the levels of fecal BSs are higher in the week preceding NEC than in controls. Serial measurement of fecal BSs may help to identify preterm infants who are vulnerable to developing NEC.

## Supporting Information

S1 File Patient characteristics. (SAV)

S2 File Individual fecal bile salts_conjugated and unconjugated. (SAV)

S3 File Total unconjugated fecal bile salts_three time points. (SAV)

## References

[1] Neu J, Walker WA. Necrotizing enterocolitis. N Engl J Med. 2011 1 20;364(3):255–64. 10.1056/NEJMra1005408 21247316 PMC3628622

[2] Watkins JB, Szczepanik P, Gould JB, Klein P, Lester R. Bile salt metabolism in the human premature infant. Preliminary observations of pool size and synthesis rate following prenatal administration of dexamethasone and phenobarbital. Gastroenterology. 1975 9;69(3):706–13. 1158088

[3] Hammons JL, Jordan WE, Stewart RL, Taulbee JD, Berg RW. Age and diet effects on fecal bile acids in infants. J Pediatr Gastroenterol Nutr. 1988 Jan-Feb;7(1):30–8. 3335983 10.1097/00005176-198801000-00008

[4] Boehm G, Braun W, Moro G, Minoli I. Bile acid concentrations in serum and duodenal aspirates of healthy preterm infants: effects of gestational and postnatal age. Biol Neonate. 1997;71(4):207–14. 9129789 10.1159/000244419

[5] Halpern MD, Dvorak B. Does abnormal bile acid metabolism contribute to NEC? Semin Perinatol. 2008 4;32(2):114–21. 10.1053/j.semperi.2008.01.005 18346535 PMC2329917

[6] Halpern MD, Weitkamp JH, Mount Patrick SK, Dobrenen HJ, Khailova L, Correa H, et al Apical sodium-dependent bile acid transporter upregulation is associated with necrotizing enterocolitis. Am J Physiol Gastrointest Liver Physiol. 2010 9;299(3):G623–31. 10.1152/ajpgi.00242.2010 20616306 PMC2950692

[7] Martin NA, Mount Patrick SK, Estrada TE, Frisk HA, Rogan DT, Dvorak B, et al Active transport of bile acids decreases mucin 2 in neonatal ileum: implications for development of necrotizing enterocolitis. PLoS One. 2011;6(12):e27191 10.1371/journal.pone.0027191 22162748 PMC3230578

[8] Corpeleijn WE, de Waard M, Christmann V, van Goudoever JB, Jansen-van der Weide MC, Kooi EM, et al Effect of Donor Milk on Severe Infections and Mortality in Very Low-Birth-Weight Infants: The Early Nutrition Study Randomized Clinical Trial. JAMA Pediatr. 2016 5 2. 10.1001/jamapediatrics.2016.0183 27135598

[9] van Meer H, Boehm G, Stellaard F, Vriesema A, Knol J, Havinga R, et al Prebiotic oligosaccharides and the enterohepatic circulation of bile salts in rats. Am J Physiol Gastrointest Liver Physiol. 2008 2;294(2):G540–7. 10.1152/ajpgi.00396.2007 18079281

[10] Inoue T, Kimura A, Aoki K, Tohma M, Kato H. Developmental pattern of 3-oxo-delta 4 bile acids in neonatal bile acid metabolism. Arch Dis Child Fetal Neonatal Ed. 1997 7;77(1):F52–6. 9279184 10.1136/fn.77.1.f52 PMC1720670

[11] Sharp HL, Peller J, Carey JB Jr, Krivit W. Primary and Secondary Bile Acids in Meconium. Pediatr Res 1971;5: 274–279.

[12] Watkins JB, Järvenpää AL, Szczepanik-Van Leeuwen P, Klein PD, Rassin DK, Gaull G, et al Feeding the low-birth weight infant: V. Effects of taurine, cholesterol, and human milk on bile acid kinetics. Gastroenterology. 1983 10;85(4):793–800. 6884704

[13] Scholmerich J, Becher MS, Schmidt K, Schubert R, Kremer B, Feldhaus S, et al Influence of hydroxylation and conjugation of bile salts on their membrane-damaging properties: studies on isolated hepatocytes and lipid membrane vesicles. Hepatology 1984 Jul-Aug;4:661–6. 6745854 10.1002/hep.1840040416

[14] Halpern MD, Holubec H, Saunders TA, Dvorak K, Clark JA, Doelle SM, et al Bile acids induce ileal damage during experimental necrotizing enterocolitis. Gastroenterology. 2006 2;130(2):359–72. 10.1053/j.gastro.2005.10.023 16472592 PMC3417808

[15] Out C, Patankar JV, Doktorova M, Boesjes M, Bos T, de Boer S, et al Gut microbiota inhibit Asbt-dependent intestinal bile acid reabsorption via Gata4. J Hepatol. 2015 9;63(3):697–704. 10.1016/j.jhep.2015.04.030 26022694 PMC5293168

[16] Gephart SM, Spitzer AR, Effken JA, Dodd E, Halpern M, McGrath JM. Discrimination of GutCheck(NEC): a clinical risk index for necrotizing enterocolitis. J Perinatol. 2014 6;34(6):468–75. 10.1038/jp.2014.37 24651734 PMC4420242

[17] Schurink M, Kooi EM, Hulzebos CV, Kox RG, Groen H, Heineman E, et al Intestinal Fatty Acid-binding protein as a diagnostic marker for complicated and uncomplicated necrotizing enterocolitis: a prospective cohort study. PLoS One. 2015 3 20;10(3):e0121336 10.1371/journal.pone.0121336 25793701 PMC4368100

